# A rare presentation: Morel-Lavallée lesion of the lateral thigh

**DOI:** 10.1093/jscr/rjag603

**Published:** 2026-07-18

**Authors:** Muhammad A Rafay, Anirudh Mathur, Robert Manton, Benjamin Smeeton, Bhagwat Mathur

**Affiliations:** Barts and The London School of Medicine and Dentistry, Queen Mary University of London, London, E1 2AD, United Kingdom; School of Medicine, New Vision University, Tbilisi, 0158, Georgia; St Andrew's Centre for Plastic Surgery and Burns Mid Essex Hospital Services NHS Trust, Essex, CM1 7ET, United Kingdom; St Andrew's Centre for Plastic Surgery and Burns Mid Essex Hospital Services NHS Trust, Essex, CM1 7ET, United Kingdom; St Andrew's Centre for Plastic Surgery and Burns Mid Essex Hospital Services NHS Trust, Essex, CM1 7ET, United Kingdom

**Keywords:** Morel-Lavallée lesion, closed degloving injury, pseudocapsule formation, surgical capsulectomy, chronic seroma

## Abstract

Morel-Lavallée lesions (MLLs) are rare, closed degloving injuries resulting from trauma that detaches subcutaneous tissue from the underlying fascia, resulting in collections of haemolymphatic fluid. Late diagnosis can lead to pseudocapsule formation, chronic pain, and physical impairment. We describe an unusual case of a large persistent MLL on the outer aspect of the thigh that presented several years following a low-energy injury, having been initially misdiagnosed as a haematoma. Gradual expansion resulted in chronic pain and significant functional limitations. Magnetic resonance imaging revealed a substantial encapsulated subcutaneous mass consistent with a chronic MLL. Management was attained via open surgical excision with capsulectomy, quilting sutures, and closed-suction drainage. The patient experience full symptom resolution and no recurrence at follow up. This case emphasizes the need for increasing awareness amongst clinicians and demonstrates that prompt imaging and surgical treatment can avert long-term complications.

## Introduction

Morel-Lavallée lesions (MLLs) are infrequent closed degloving injuries. They are caused by tangential shearing forces that damage perforating blood vessels and lymphatics, leading to a build-up of haemolymphatic fluid in the potential space between subcutaneous tissue and deep fascia [1, 2]. They usually occur over the greater trochanter, pelvis, and thigh after blunt trauma [3]. Acute lesions can respond well to conservative treatment; however, a delayed or missed diagnosis may result in pseudocapsule formation and chronic seroma development, which makes non-operative therapies ineffective and raises the likelihood of recurrence [4–6]. We describe an unusual instance of a large chronic MLL of the lateral thigh, characterized by an extended duration of symptoms and considerable functional effects.

## Case presentation

A 67-year-old woman was sent for evaluation due to a swelling on her left lateral hip that had been gradually increasing in size. The mass appeared following a low-speed mechanical fall onto her garden step in September 2022. It was initially diagnosed as a haematoma. Despite conservative treatment, the swelling persisted and increased in size, leading to chronic pain, compromised balance, challenges with clothing and sleep, and eventual reliance on a wheelchair.

Upon presentation to our clinic some three years following the trauma, a sizeable, solid, well-defined mass ~30 cm in diameter was observed over the left greater trochanter (Fig. 1). No indications of infection were present. Her past medical history included hypertension, microvascular angina, gastroesophageal reflux disease, along with anxiety and depression. Her body mass index was 41.2 kg/m^2^.

**Figure 1:**
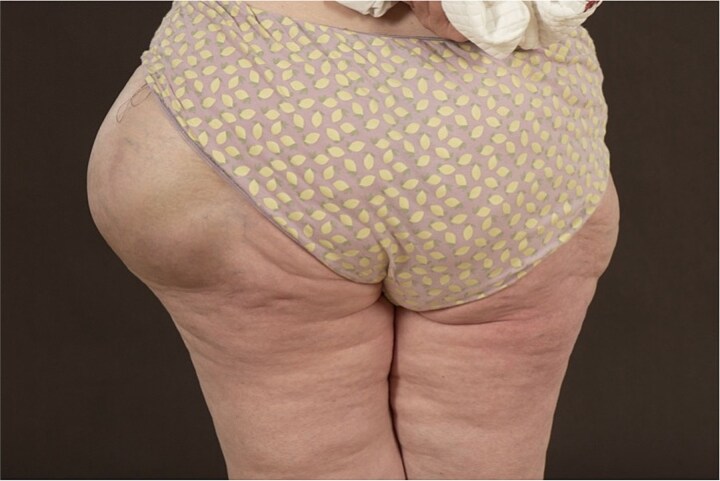
Clinical photograph demonstrating a large, well-circumscribed swelling over the left greater trochanter.

Subsequent magnetic resonance imaging (MRI) demonstrated a well-defined subcutaneous fluid collection lateral to the left greater trochanter. On axial imaging, the lesion measured ~15.7 cm anteroposteriorly and 10.8 cm transversely, with heterogeneous internal signal consistent with chronic blood products and fibrinous debris. A low-signal peripheral rim suggested pseudocapsule formation (Fig. 2) [3, 5].

**Figure 2:**
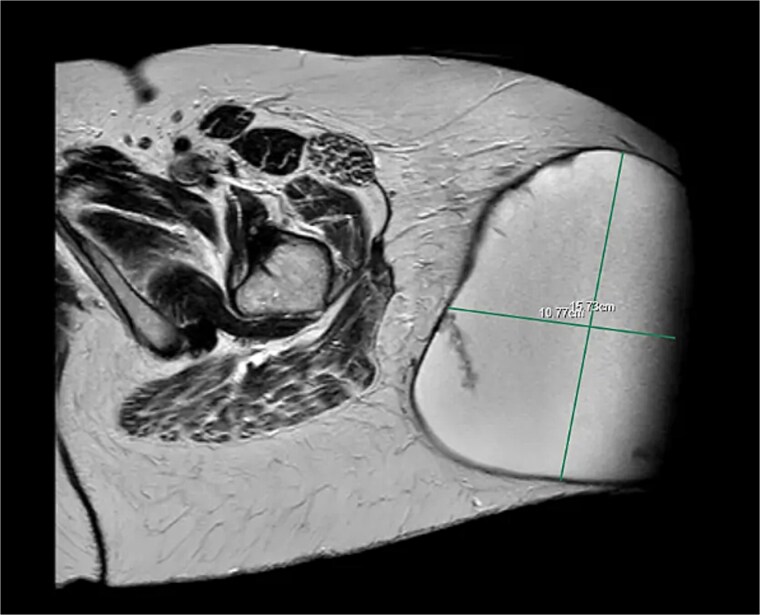
Axial MRI demonstrating a large, encapsulated subcutaneous fluid collection lateral to the left greater trochanter, consistent with a chronic MLL.

Inferiorly, the lesion remained superficial to the fascia lata, tapering to ~7.9 × 10.9 cm, with internal hypointense foci consistent with fat globules or organizing thrombus (Fig. 3) [5, 6].

**Figure 3:**
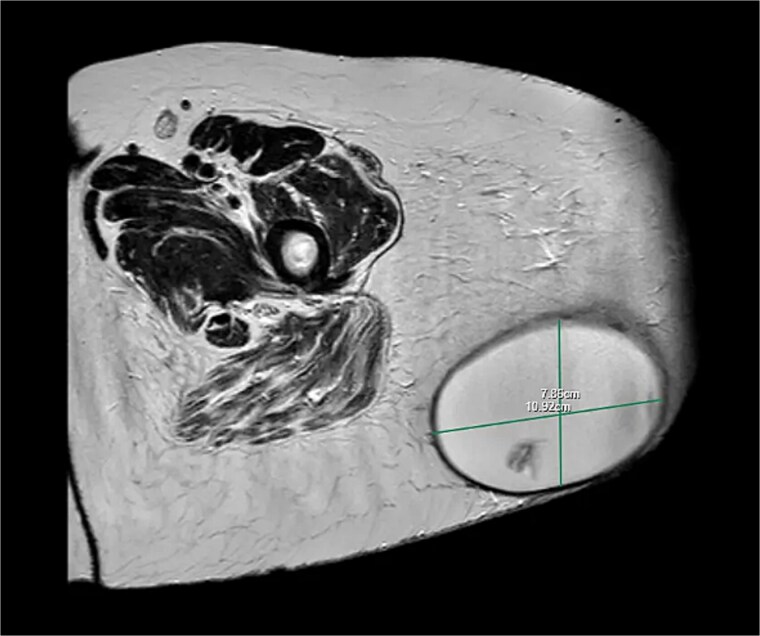
Axial MRI at a more distal level demonstrating the lesion remaining superficial to the fascia lata with heterogeneous internal signal.

Given the lesion’s size, chronicity, and severe functional impairment, surgical excision was planned and expedited. In October 2025, the patient underwent open excision under general anaesthesia. A large, encapsulated seroma was excised in its entirety without rupture (Fig. 4). Due to mechanical stretch of the overlying skin, a skin ellipse was excised. In addition, quilting sutures were used to obliterate dead space, and a low vacuum suction drain was placed, consistent with recommended surgical principles [7, 8].

**Figure 4:**
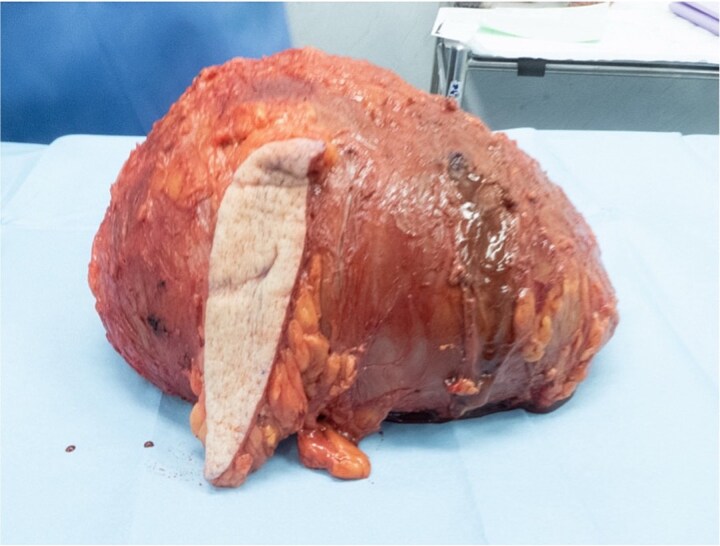
Intra-operative photograph demonstrating the excised encapsulated MLL following complete capsulectomy.

Histological analysis demonstrated a chronic organizing seroma measuring 230 × 250 × 100 mm with a fibrous capsule ~3 mm thick. Microscopy showed fibrous connective tissue with haemosiderin-laden macrophages, cholesterol clefts, and fibrinous debris, with no evidence of malignancy or infection.

At follow-up at 8 weeks, the surgical site had healed well with no evidence of recurrence (Fig. 5). The patient reported complete resolution of pain and mechanical symptoms, with restoration of mobility and independence.

**Figure 5:**
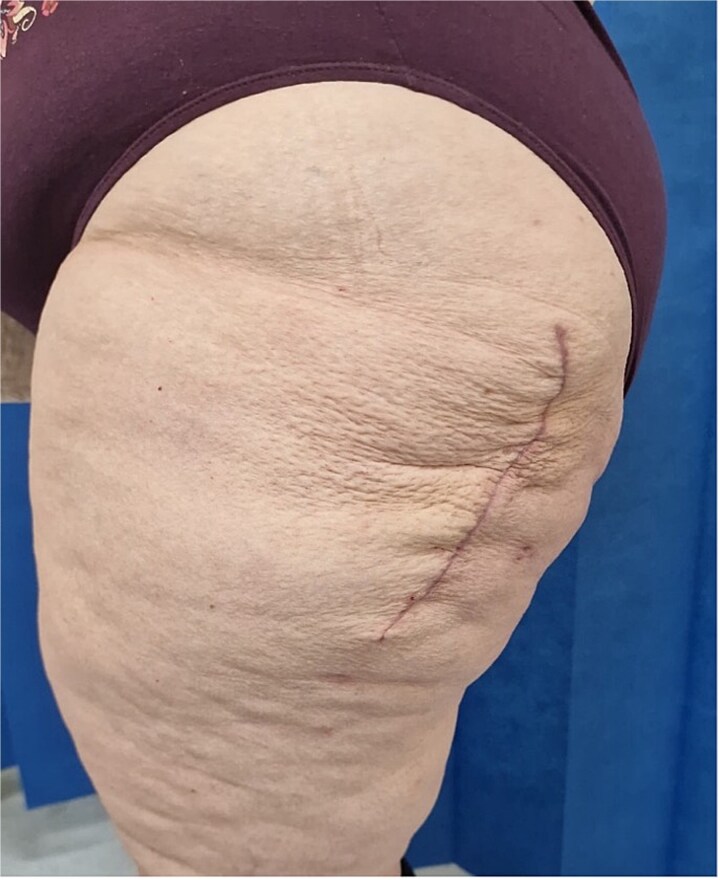
Eight-week post-operative follow up photograph demonstrating satisfactory wound healing with no evidence of recurrence.

## Discussion

This case presentation is notable for the extreme size and chronicity of the lesion following such low-energy trauma, resulting in profound functional impairment that was fully reversed following definitive surgical excision.

MLLs are often incorrectly diagnosed as post-traumatic haematomas, especially when their presentation is delayed or linked to low energy trauma [4, 9]. In contrast to haematomas, MLLs are caused by shearing forces that form a lasting potential space susceptible to continuous lymphatic leakage and gradual expansion [1, 3]. Not acknowledging this difference can postpone conclusive treatment and increase morbidity.

MRI is the preferred imaging technique, enabling precise evaluation of lesion size, internal features, and pseudocapsule development, all of which affect treatment [3–5]. Chronic lesions usually exhibit a distinctly defined capsule with varied internal signals resulting from blood breakdown products and necrotic adipose tissue [5, 6]. The Mellado–Bencardino classification offers a valuable structure for assessing lesions and informing treatment choices (Table 1) [3, 10].

**Table 1:** MRI classification of MLLs according to Mellado–Bencardino [10].

Lesion type	Morphology	T1 weighted signal	T2 weighted signal	Other key features
Type 1—Seroma	Laminar	Homogenously hypointense	Hyperintense	No outer capsule formation
Type 2—Subacute haematoma	Oval	Homogenously hyperintense	Hyperintense	Presence of methaemoglobin, thin capsule formation
Type 3—Chronic organizing haematoma	Oval	Hypointense	Heterogenous hypointense/isointense	Thich capsule formation, capsular and internal enhancement on post contrast sequence
Type 4—Closed laceration	Linear	Hypointense	Hyperintense	No capsule formation
Type 5—Small rounded pseudonodular lesion	Round	Variable	Variable	Variable capsule formation
Type 6—Superimposed infection	Variable +/− sinus tract	Variable	Variable	Thick enhancing capsule

Management is influenced by the size of the lesion and how long it has been present. Minor lesions (Type 1 and Type 4) may improve with compression or aspiration. Type 2 lesions may initially be managed similarly, although recurrence following aspiration alone is common, with reported rates exceeding 50% [11]. Sclerotherapy, especially with doxycycline, has demonstrated potential in specific chronic cases (such as Type 3 lesions), yet the evidence is still scarce and varied [12, 13]. For extensive, long-standing, encapsulated lesions (Type 3 and Type 6), open surgical excision accompanied by capsulectomy and careful management of dead space continues to be the most dependable treatment, lowering recurrence rates to around 15%–20% [7, 8, 11, 14].

## Conclusion

Ongoing post traumatic soft tissue swelling should raise suspicion of a MLL, especially if symptoms worsen or do not improve. MRI is vital for diagnosing and staging. Persistent, large lesions with pseudocapsule development should not be expected to improve with conservative treatments alone and should be treated surgically. Prompt identification and action can avert chronic sequelae and greatly enhance patient recovery.
